# Characteristics and clinical significance of immune cells in omental milky spots of patients with gastric cancer

**DOI:** 10.3389/fimmu.2025.1521278

**Published:** 2025-01-30

**Authors:** Yasunobu Mano, Yuka Igarashi, Keisuke Komori, Itaru Hashimoto, Hayato Watanabe, Kosuke Takahashi, Kazuki Kano, Hirohito Fujikawa, Takanobu Yamada, Hidetomo Himuro, Taku Kouro, Feifei Wei, Kayoko Tsuji, Shun Horaguchi, Mitsuru Komahashi, Takashi Oshima, Tetsuro Sasada

**Affiliations:** ^1^ Division of Cancer Immunotherapy, Kanagawa Cancer Center Research Institute, Yokohama, Japan; ^2^ Cancer Vaccine and Immunotherapy Center, Kanagawa Cancer Center Research Institute, Yokohama, Japan; ^3^ Department of Gastrointestinal Surgery, Kanagawa Cancer Center, Yokohama, Japan; ^4^ Department of Surgery, Yokohama City University, Yokohama, Japan; ^5^ Department of Pediatric Surgery, Nihon University School of Medicine, Tokyo, Japan

**Keywords:** omental milky spots, gastric cancer, adaptive immune responses, lymphatic invasion, immune microenvironment

## Abstract

The omentum is a common site of peritoneal metastasis in various cancers, including gastric cancer. It contains immune cell aggregates known as milky spots, which provide a microenvironment for peritoneal immunity by regulating innate and adaptive immune responses. In this study, we investigated gene expression profiles in cells from omental milky spots of patients with gastric cancer (n = 37) by RNA sequencing analysis and classified the patients into four groups (G1-4). Notably, significant differences were observed between the groups in terms of macroscopic type, lymphatic invasion, venous invasion, and pathological stage (pStage). G3, which was enriched in genes related to acquired immunity, showed earlier tumor stages (macroscopic type 0, Ly0, V0, and pStage I) and a better prognosis. In contrast, G4 showed enrichment of genes related to neutrophils and innate immunity; G1 and G2 showed no enrichment of innate or adaptive immune-related genes, suggesting an immune desert microenvironment. Cytometric analysis revealed significantly more T and B cells and fewer neutrophils in G3. Accordingly, the immune microenvironment in omental milky spots may vary depending on the stage of gastric cancer progression. When univariate Cox proportional hazards regression models were used to search for prognostically relevant genes specific to G3, 23 potential prognostic genes were identified as common genes associated with relapse-free survival and overall survival. In addition, the multivariate Cox proportional hazards model using these prognostic genes and clinicopathological information showed that combining the B cell marker *CD19* and Ly had a high predictive accuracy for prognosis. Based on this study’s results, it is possible that tumor progression, such as lymphatic and/or venous infiltration of tumor cells, may affect the immune cell composition and proportions in omental milky spots of patients with gastric cancer and analysis of gene expression in omental milky spots may help to predict gastric cancer prognosis.

## 1 Introduction

According to the 2020 Global Cancer Statistics, gastric cancer is the fifth most common cancer worldwide, affecting over 1 million people annually, and is the fourth leading cause of cancer-related deaths. Especially in East Asia, the incidence of gastric cancer is significantly higher due to the prevalence of *Helicobacter pylori* infection and the East Asian strain-specific CagA harbored by *Helicobacter pylori* as a carcinogenic factor (1–3). Despite the recent advances in diagnostic and therapeutic techniques for gastric cancer, some estimates suggest that the incidence of gastric cancer will increase to 1.8 million cases by 2040 (4).

The omentum, which is primarily surrounded by fatty tissue, is a common site of peritoneal metastasis in various cancers, such as gastric, colorectal, and ovarian cancers (5–7). Peritoneal metastases occur in approximately 10–20% of patients with gastric cancer and result in very poor prognosis (8–15), but their mechanisms remain unclear. Many researchers have focused on omental milky spots, a secondary lymphoid organ with unique structural and functional features (16–19), as a site of peritoneal seeding of cancer cells. Omental milky spots contribute to peritoneal immunity by supporting innate and adaptive immune responses through collecting antigens, particulates, and pathogens from the peritoneal cavity and regulating various immune responses, including inflammation and tolerance, in response to their stimuli (5, 8, 20–23). In addition, the milky spots are highly efficient “natural filters” for sorting cancer cells and provide a microenvironment in which cancer cells can proliferate by inducing angiogenesis and evading immune responses, leading to the formation of metastatic nests (24–28). Moreover, the large number of adipocytes surrounding the milky spot may provide lipids to meet the energy needs of cancer cells through their lipolysis and β-oxidation, thereby promoting the proliferation of adherent cancer cells (29–31).

Omental milky spots are composed of various immune cells such as T, B, and natural killer cells, and macrophages. In the human fetal and mouse milky spots, B cells occupy the majority of lymphocytes (17, 21); whereas in adult humans, T cells comprise the majority of lymphocytes (32, 33). In a study comparing the omental milky spots between gastric and rectal cancers, the number of B lymphocytes was higher in rectal cancer, suggesting that the proportions of cells constituting the omental milky spots seem to vary significantly depending on the cancer type. Although several reports have described the immunological characteristics of the omental milky spot in ovarian and colorectal cancers (6, 7, 34–36), few studies have reported these characteristics in gastric cancer. Therefore, we aimed to characterize the gene expression profiles of cells from the omental milky spots of 37 patients with gastric cancer and classified them into four groups. We also aimed to examine the possible correlations between these gene expression profiles and the clinical characteristics and outcomes of patients with gastric cancer.

## 2 Methods

### 2.1 Clinical samples

We enrolled 37 patients who underwent gastrectomy for various stages of gastric cancer at Kanagawa Cancer Center (Yokohama, Japan) between March and December 2020. Patients with multiple cancers or other serious comorbidities such as liver, kidney, infectious, and autoimmune diseases were excluded. The study was conducted in accordance with the provisions of the Declaration of Helsinki and was approved by the Institutional Review Board of Kanagawa Cancer Center (approval number: 2019-134). After explaining the nature and possible outcomes of the study, written informed consent was obtained from all participants before inclusion in this study.

### 2.2 Immune cell preparation

The omentum was resected during gastrectomy. The milky spots isolated from the resected omentum with a scalpel or the omental tissues near the branches of the gastroepiploic vessels were washed well with phosphate-buffered saline (Gibco, Life Technologies, NY, USA), and enzymatically digested by incubation with 0.08% collagenase I (STEMCELL Technologies, Vancouver, Canada) in phosphate buffered saline containing 1% fetal bovine serum (Hyclone, Logan, USA) at 37°C for 90 min. Reportedly, omental milky spots have been identified macroscopically as cotton-wool-like structures located near the first or second branches of the gastroepiploic vessels (37); however, it was often difficult to readily identify them by examining fresh omentum due to the lack of clear macroscopic characteristics or distinct color. In such cases, cells were purified from omental tissues near the branches of the gastroepiploic vessels, expected to contain omental milky spots. After passing through a 100-μm cell strainer (Corning, Arizona, USA), the cells were collected by centrifugation at 1000 × *g* for 10 min at 4°C. Afterwards, the cells were suspended with ammonium–chloride–potassium lysis buffer (Gibco, Life Technologies, NY, USA) for 5 min at 4°C, centrifuged at 400 × *g* for 5 min at 4°C, and frozen until further analysis. Peripheral blood mononuclear cells (PBMCs) were purified from peripheral blood by Ficoll-Paque Plus (GE Healthcare, Uppsala, Sweden) density centrifugation and frozen until further analysis.

### 2.3 RNA sequencing

The cells collected from the omental milky spots were sent to Beijing Genomics Institute (BGI, Shenzhen, China), where RNA sequencing (RNA-Seq) was performed on the DNBSEQ PE100 platform, a high-throughput DNA sequencing system. RNA was extracted at BGI with TRIzol (Invitrogen, CA, USA), and RNA quality was assessed on an Agilent 2100 Bioanalyzer (Agilent Technologies, CA, USA). RNA-Seq results detected more than 40 million reads in each sample. The quality control and filtering of the RNA-Seq fastq files were performed using fastp (38). The RNA-Seq files were then aligned to GRCh38 (GENCODE v42) using STAR (2.7.10b) (39) and at least 90% of each sample was mapped. Accurate normalization and quantification of the gene expression levels (transcripts per million [TPM] and count) were calculated using RSEM (v1.3.3) (40). Gene ontology (GO) analysis was performed using Metascape (https://metascape.org/) (41). The differentially expressed genes (DEGs) were calculated from the count data using DESeq2 (v1.38.3) (42). The following criteria: abs (log_2_FC) ≥ 1, *q* < 0.05, *p* < 0.05 were considered as DEGs. Heatmaps were generated from TPM data converted to z-score and displayed using ComplexHeatmap (43). Enrichment map analysis was analyzed and displayed using clusterProfiler (44). RNA-Seq data of PBMCs from healthy subjects in public databases (GSM2859500-2859505, GSM2859531-2859537) were used as reference data (45).

### 2.4 Hierarchical clustering

The genes on the X and Y chromosomes were excluded from the TPM data from the RNA-Seq of 37 samples, and 7,060 genes with variable expression in each sample were extracted using a standard deviation (SD) value > 3. Hierarchical clustering analysis was performed using Cluster 3.0 (46) and displayed using Java TreeView (47).

### 2.5 Gene set enrichment analysis

Gene set enrichment analysis (GSEA) was performed using GSEA (v4.3.2; https://www.gsea-msigdb.org/gsea/index.jsp) (48). We used 50 hallmark and 196 pathway interaction database (PID) gene sets, respectively, for the GSEA. Characteristic gene sets for each subgroup were extracted under the conditions of abs (NES) ≥ 1.5 and MIN_FDR < 0.05 in all the subgroups. For the GSEA of Canonical pathways and cell type signatures, the top 20 gene sets in each group were selected out of 3917 and 830 gene sets, respectively, and duplicates were excluded.

### 2.6 Digital cytometry

We used two different programs, CIBERSORTx and MCP-Counter, to estimate the proportions of different immune cell subsets (49, 50). For CIBERSORTx, the TPM data from bulk RNA-Seq were analyzed using the leukocyte signature matrix (LM22). For MCP-Counter, the percentages of 10 types of immune cells were estimated from the log_2_(TPM+1) data of the bulk RNA-Seq.

### 2.7 Cell type signature genes

For adipocytes, mesothelial cells, T cells, and neutrophils, we used the top 25 signature genes as previously reported (51). For the epithelial cells, we used the most frequently duplicated genes among the signature genes that are registered in CellMarker 2.0 (52) (http://bio-bigdata.hrbmu.edu.cn/CellMarker or http://117.50.127.228/CellMarker/).

### 2.8 Flow cytometry

Flow cytometry data were acquired using BD FACSDiva software (v.9.0; Becton Dickinson, CA, USA) on a FACSCanto II flow cytometer (BD Bioscience) and were analyzed using FlowJo (v.7.6.5; Tree Star, OR, USA). For the analysis of the surface markers, the cells were stained with specific antibodies in phosphate buffered saline containing 2% fetal bovine serum for 30 min at 4°C. Next, 7-AAD (A0770400, 1:20, Beckman) was used for dead-cell exclusion. The following antibodies from BioLgend were used: Fluorescein Isothiocyanate (FITC)–anti-cluster of differentiation (CD)3 (UCHT1, 300406, 1:20); Phycoerythrin (PE)–anti-CD56 (HCD56, 318306, 1:20); Allophycocyanin (APC)–anti-CD8 (RPA-T8, 301014, 1:80); PE/Cyanine 7–anti-CD4 (OKT4, 317414, 1:80); FITC–anti-CD19 (HIB19, 302206, 1:20); APC–anti-γ/δ T cell receptor (TCR) (B1, 331212, 1:20); PE/Cyanine 7–anti-CD27 (M-T271, 356412, 1:20); APC/Cyanine 7–anti-immunoglobin D (IA6-2, 348218, 1:20); FITC–anti-CD8 (HIT8a, 300906, 1:80); APC/Cyanine 7–anti-CD45RA (HI100, 304128, 1:20); Alexa Fluor 488–anti-CCR7 (G043H7, 353206, 1:20); PE–anti-CD33 (WM53, 303404, 1:20); APC–anti-CD11b (ICRF44, 301310, 1:20); and APC/Cyanine 7–anti-CD14 (HCD14, 355620, 1:20).

### 2.9 Statistical analyses

All statistical analyses were performed using the R software package (version 4.2.1; http://www.r-project.org). Correlations of the binary outcome variables of clinical information were checked using Fisher’s exact test. Normal distribution was assessed using the non-parametric Shapiro–Wilk test. Equal variances were evaluated using Bartlett’s test. For the data having normal distribution and equal variances, an analysis of variance was performed. If significant differences were obtained, the Tukey–Kramer test was used to determine which pairwise comparisons were significant. For the data having non-normal distribution or unequal variances, Kruskal–Wallis test was performed. If a significant difference was obtained, the Steel–Dwass test was used to determine which pairwise comparisons were significant. Survival analysis was performed till March 31, 2023. Overall survival (OS) and relapse-free survival (RFS) were analyzed using the Kaplan–Meier method, and *p*-values for between-group comparisons were calculated using the log-rank test. Univariate Cox proportional hazards regression was used to explore potential prognostic factors, with TPM ≥ median of gene expression as high expression and TPM < median as low expression. For the multivariate Cox proportional hazards model, two variables were selected taking into account the sample size. *P*-values < 0.05 were considered statistically significant.

## 3 Results

### 3.1 Patients with gastric cancer were classified into four groups based on the transcriptomic analysis of the cells in the omental milky spots

**Figure 1:**
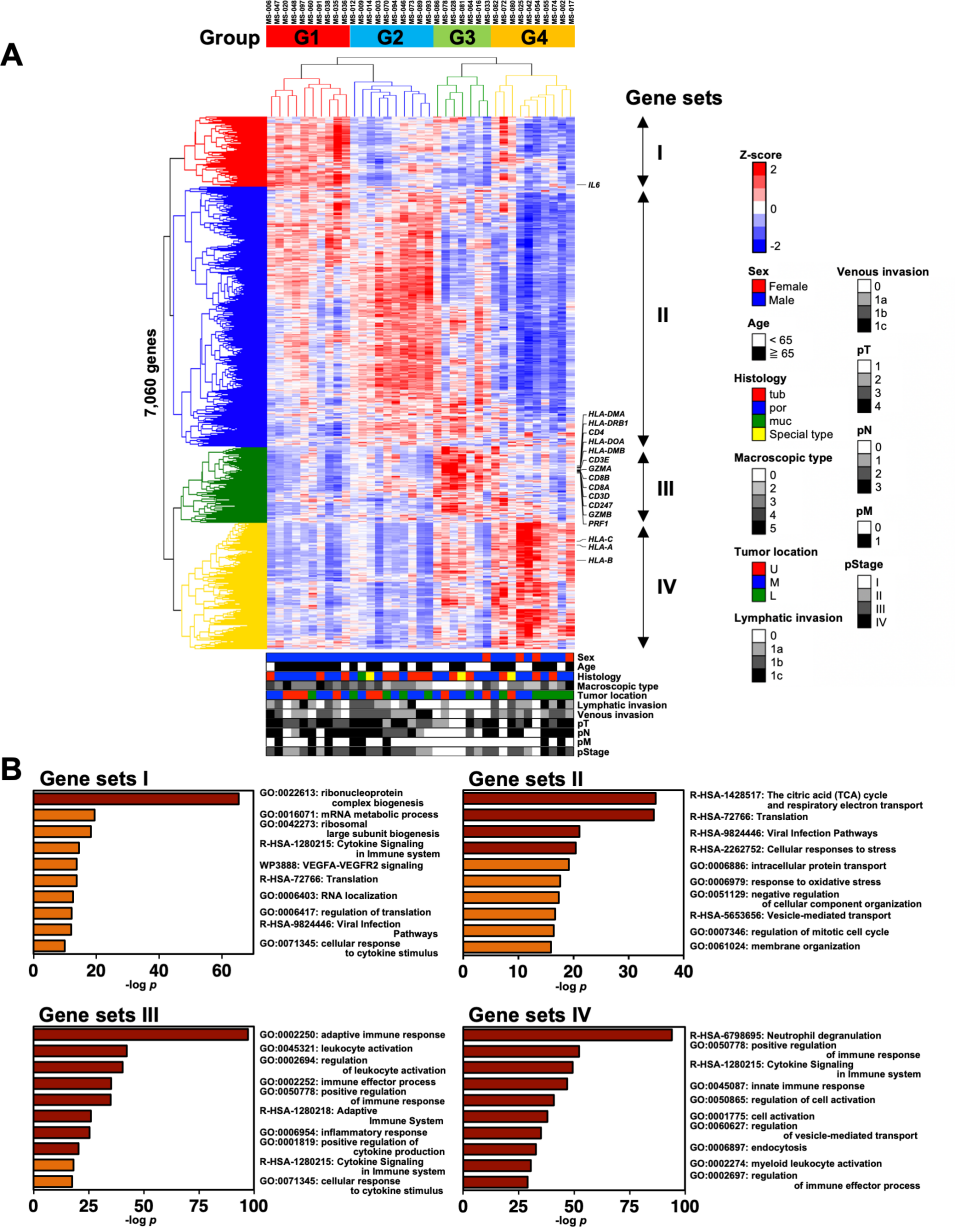
Classification of patients with gastric cancer into four groups by hierarchical clustering analysis with the transcriptomic data of cells in the omental milky spots. **(A)** Unsupervised two-way hierarchical clustering was performed on 7,060 genes of RNA-Seq data of cells in the omental milky spots of 37 patients with gastric cancer. The horizontal axis shows each case, and the vertical axis shows each gene. The clinical information data are shown in the bottom row as follows: sex, age, histology, macroscopic type, tumor location, lymphatic invasion, venous invasion, pT, pN, pM, and pStage. **(B)** Gene ontology analysis was performed on gene sets that were characteristic of each group, and the top 10 terms are shown.

We performed RNA sequencing (RNA-Seq) analysis of the cells derived from the omental milky spots in 37 patients with gastric cancer to clarify the characteristics of the cells. We first extracted 7,060 distinct genes from the RNA-Seq data and performed hierarchical clustering analysis to determine which gene expression features characterize the transcriptome of the cells in the omental milky spots. The gene expression status was classified into four groups named G1–4 (**Figure 1A**). **Figure 1B** shows the GO analysis of gene sets I–IV, which are the characteristic genes in each of the four groups. G1 and G2 were enriched in terms related to ribosomes and citric acid cycle, respectively. Interestingly, G3 was enriched in terms related to the adaptive immune response (*p* = 6.6×10^-98^), whereas G4 was enriched in those related to neutrophil degranulation (*p* = 1.0×10^-96^) and innate immune response (*p* = 1.4×10^-47^).

We also analyzed the DEGs in each group using DESeq2 to validate the hierarchical clustering analysis results (**Supplementary Figure 1**). The numbers of DEGs in each group classified by the clustering analysis were higher than those from the clinical characteristics, such as Ly, pN, and pM (**Supplementary Figure 1A**). The DEGs for each group (vs. the others) were enriched with similar terms as those of the GO analysis, consistently validating the hierarchical clustering analysis results (**Figure 1**, **Supplementary Figures 1B, C**).

### 3.2 Clinical features and prognosis in the four groups classified using transcriptomic analysis

We statistically analyzed the clinical information in each group to explore the features of the four transcriptomic groups. Multiple comparisons revealed no significant differences with respect to sex, age, histology, tumor location, pT, pN, and pM (**Table 1**). In contrast, significant differences were observed in the macroscopic type (*p* = 0.0072), lymphatic invasion (Ly, *p* = 0.0358), venous invasion (V, *p* = 0.0042), and pathological stage (pStage, *p* = 0.0026) (**Figure 1A**, **Table 1**). Pairwise comparisons revealed significant differences between G3 and the other groups in terms of macroscopic type (*p* = 0.0003), Ly (*p* = 0.0051), V (*p* = 0.0049), and pStage (*p* = 0.0068); G3 was significantly more likely to have macroscopic type 0, Ly0, V0, and pStage I (**Table 2**).

**Table 1:** Clinical characteristics in four transcriptomic groups in the patients with gastric cancer.

Clinical characteristics	All cases	G1 group	G2 group	G3 group	G4 group	Multiple Comparisons
	Number (%), Median (±SD)					*p*-value
Number of patients	37 (100.0%)	10 (27.0%)	10 (27.0%)	7 (18.9%)	10 (27.0%)	
Sex						
Male	33 (89.2%)	10 (100.0%)	10 (100.0%)	6 (85.7%)	7 (70.0%)	0.0641
Female	4 (10.8%)	0 (0.0%)	0 (0.0%)	1 (14.3%)	3 (30.0%)	
Age (years)						
Median (SD)	71.0 (±12.1)	77.5 (±11.7)	69.5 (±12.4)	63.0 (±11.1)	70.5 (±12.9)	0.2880
Histology						
tub	13 (35.1%)	3 (30.0%)	5 (50.0%)	2 (28.6%)	3 (30.0%)	0.7149
por	20 (54.1%)	7 (70.0%)	3 (30.0%)	4 (57.1%)	6 (60.0%)	
muc	1 (2.7%)	0 (0.0%)	1 (10.0%)	0 (0.0%)	0 (0.0%)	
Special type	3(8.1%)	0 (0.0%)	1 (10.0%)	1 (14.3%)	1 (10.0%)	
Macroscopic type						
0	6 (16.2%)	0 (0.0%)	0 (0.0%)	5 (71.4%)	1 (10.0%)	^**^0.0072
1	0 (0.0%)	0 (0.0%)	0 (0.0%)	0 (0.0%)	0 (0.0%)	
2	7 (18.9%)	0 (0.0%)	3 (30.0%)	1 (14.3%)	3 (30.0%)	
3	14 (37.8%)	5 (50.0%)	5 (50.0%)	0 (0.0%)	4 (40.0%)	
4	7 (18.9%)	3 (30.0%)	2 (20.0%)	1 (14.3%)	1 (10.0%)	
5 (Unclassifiable)	3 (8.1%)	2 (20.0%)	0 (0.0%)	0 (0.0%)	1 (10.0%)	
Tumor location						
U (Upper)	9 (24.3%)	4 (40.0%)	2 (20.0%)	2 (28.6%)	1 (10.0%)	0.3059
M (Middle)	16 (43.2%)	5 (50.0%)	4 (40.0%)	4 (57.1%)	3 (30.0%)	
L (Lower)	12 (32.4%)	1 (10.0%)	4 (40.0%)	1 (14.3%)	6 (60.0%)	
Ly (Lymphatic invasion)						
0	14 (37.8%)	3 (30.0%)	3 (30.0%)	6 (85.7%)	2 (20.0%)	^*^0.0358
1 (1a, 1b, 1c)	23 (62.2%)	7 (70.0%)	7 (70.0%)	1 (14.3%)	8 (80.0%)	
V (Venous invasion)						
0	9 (24.3%)	3 (30.0%)	0 (0.0%)	5 (71.4%)	1 (10.0%)	^**^0.0042
1 (1a, 1b, 1c)	28 (75.7%)	7 (70.0%)	10 (100.0%)	2 (28.6%)	9 (90.0%)	
pT						
1	4 (10.8%)	0 (0.0%)	0 (0.0%)	2 (28.6%)	2 (20.0%)	0.1070
≧ 2	33 (89.2%)	10 (100.0%)	10 (100.0%)	5 (71.4%)	8 (80.0%)	
pN						
0	11 (29.7%)	2 (20.0%)	1 (10.0%)	5 (71.4%)	3 (30.0%)	0.0752
≧ 1	26 (70.3%)	8 (80.0%)	9 (90.0%)	2 (28.6%)	7 (70.0%)	
pM						
0	29 (78.4%)	7 (70.0%)	7 (70.0%)	7 (100.0%)	8 (80.0%)	0.4058
1	8 (21.6%)	3 (30.0%)	3 (30.0%)	0 (0.0%)	2 (20.0%)	
pStage						
I	6 (16.2%)	0 (0.0%)	0 (0.0%)	4 (57.1%)	2 (20.0%)	^**^0.0026
≧ II	31 (83.8%)	10 (100.0%)	10 (100.0%)	3 (42.9%)	8 (80.0%)	

For age, the multiple comparison tests with G1-4 groups were analyzed by ANOVA (analysis of variance). For sex, histology, macroscopic type, tumor location, lymphatic invasion,

venous invasion, pT, pN, pM, and pStage, the multiple comparison tests were analyzed by Fisher's exact test.

Tubular adenocarcinoma; tub, Poorly differentiated adenocarcinoma; por, Mucinous adenocarcinoma; muc. *p < 0.05, **p < 0.01. Bold text indicates statically significant.

**Table 2:** Pairwise comparisons in the clinical characteristics between the groups identified using transcriptome analysis.

Clinical characteristics	Pairwise Comparisons									
	G1 vs. G2	G1 vs. G3	G1 vs. G4	G2 vs. G3	G2 vs. G4	G3 vs. G4	G1 vs. the others	G2 vs. the others	G3 vs. the others	G4 vs. the others
	*p*-value									
Macroscopic type										
0: ≧ 1	1	^*^0.0102	1	^*^0.0102	1	0.0690	0.1621	0.1621	^***^0.0003	1
Ly (Lymphatic invasion)										
0: 1 (1a, 1b, 1c)	1	0.0996	1	0.0996	1	0.0913	0.7099	0.7099	^**^0.0051	0.2603
V (Venous invasion)										
0: 1 (1a, 1b, 1c)	0.3158	0.3069	0.6985	^*^0.0204	1	0.1035	0.6788	0.0785	^**^0.0049	0.3932
pStage										
I: ≧ II	1	^*^0.0441	0.5684	^*^0.0441	0.5684	0.3235	0.1621	0.1621	^**^0.0068	0.6527

For macroscopic type, lymphatic invasion, venous invasion, and pStage, the multiple comparison tests were analyzed by Fisher's exact test.

*p*-values were adjusted by BH method. ^*^ *p* < 0.05, ^**^ *p* < 0.01, ^***^ *p* < 0.001. Bold text indicates statically significant.

**Figure 2:**
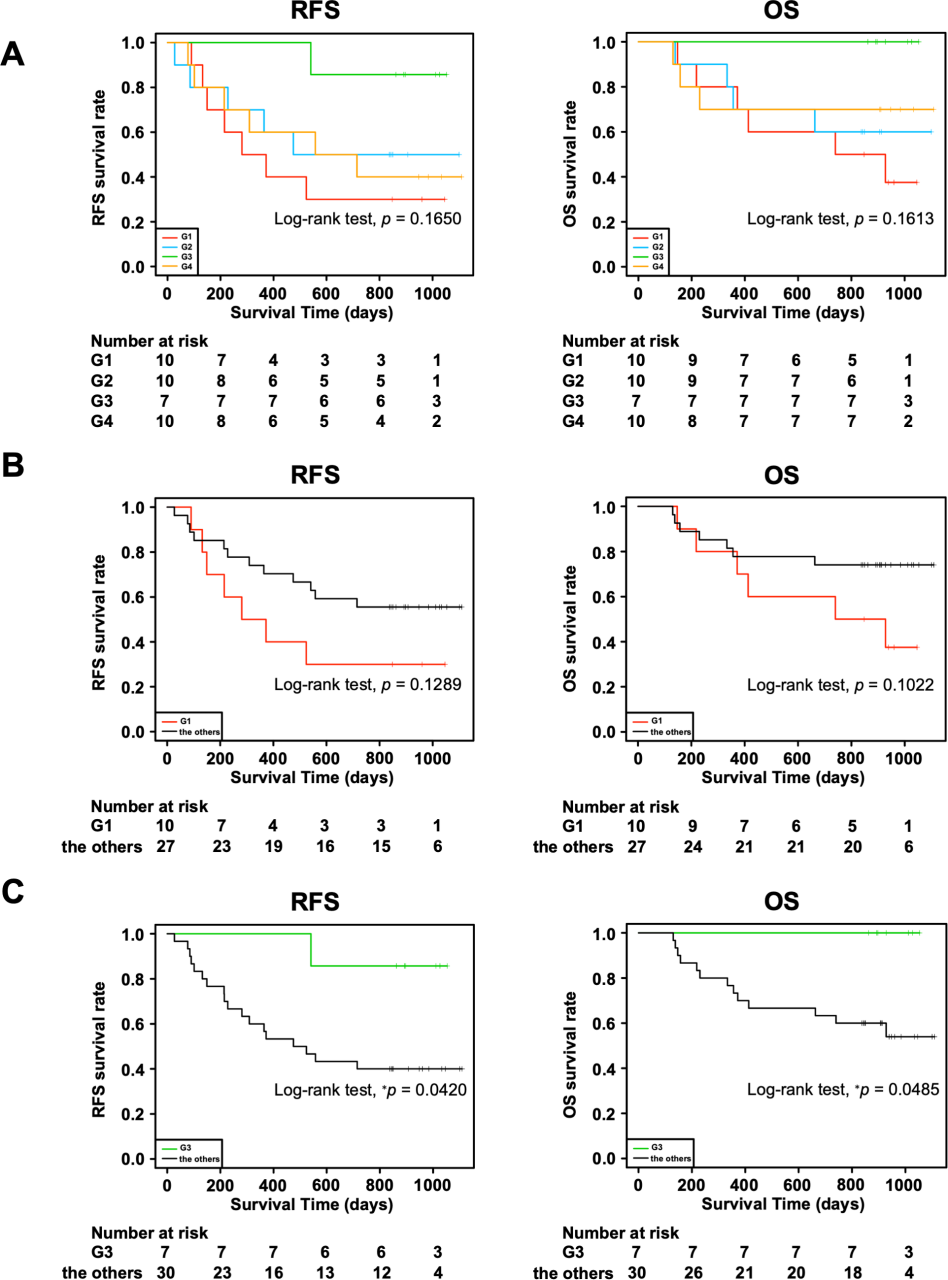
Association of four transcriptomic groups and prognosis of patients with gastric cancer. **(A)** Kaplan–Meier survival curves of recurrence-free survival (RFS) and overall survival (OS) in the four transcriptomic groups. **(B)** Kaplan–Meier survival curves of RFS and OS in the G1 group and the other groups combined. **(C)** Kaplan–Meier survival curves of RFS and OS in the G3 group and the other groups combined. *P* value was calculated using the log-rank test. ^*^ *p* < 0.05.

Moreover, we investigated the association of the four groups with RFS and OS. We observed a trend toward better prognosis in G3 and worse prognosis in G1 for both RFS and OS, although the differences were not statistically significant (**Figure 2A**: RFS, *p* = 0.1650; OS, *p* = 0.1613). In addition, there tended to be a difference in the RFS (*p* = 0.1289) and OS (*p* = 0.1022) between G1 and all the other groups combined (**Figure 2B**), whereas G3 had significantly better RFS (*p* = 0.0420) and OS (*p* = 0.0485) than all the other groups combined (**Figure 2C**). This could be partially attributed to the higher incidences of macroscopic type 0, Ly0, V0, and pStage I in the G3 group since the statistical analyses showed that these clinical characteristics tended to be associated with better RFS and OS (**Supplementary Figure 2**, **Table 3**). In addition, considering the association with the adaptive immune response in the G3 group by the GO analysis (**Figure 1B**), we speculated that the immune cells in the G3 group may contribute to a better prognosis.

**Table 3:** Survival analysis for relapse-free survival (RFS) and overall survival (OS) with clinical features in the patients with gastric cancer.

Clinical variables	RFS	OS
	*p*-value	
Sex		
Male	0.3439	0.6389
Female		
Age (years)		
< 65	0.0885	0.1275
≧ 65		
Histology		
tub	0.3525	0.0656
por		
Special type		
Macroscopic type		
0	0.0763	0.0812
≧ 1		
Tumor location		
U (Upper)	0.2882	0.6426
M (Middle)		
L (Lower)		
Ly (Lymphatic invasion)		
0	^**^0.0031	^**^0.0068
1 (1a, 1b, 1c)		
V (Venous invasion)		
0	0.0715	0.0776
1 (1a, 1b, 1c)		
pT		
1	0.2478	0.1637
≧ 2		
pN		
0	^**^0.0015	^**^0.0074
≧ 1		
pM		
0	^**^0.0088	0.2563
1		
pStage		
I	0.0763	0.0812
≧ II		

For Histology, muc was excluded from log-rank test because n = 1.

Tubular adenocarcinoma; tub, Poorly differentiated adenocarcinoma; por, Mucinous adenocarcinoma; muc.

^**^ *p* < 0.01. Bold text indicates statically significant.

### 3.3 Pathways and gene expression characteristics in the four groups classified using transcriptomic analysis

**Figure 3:**
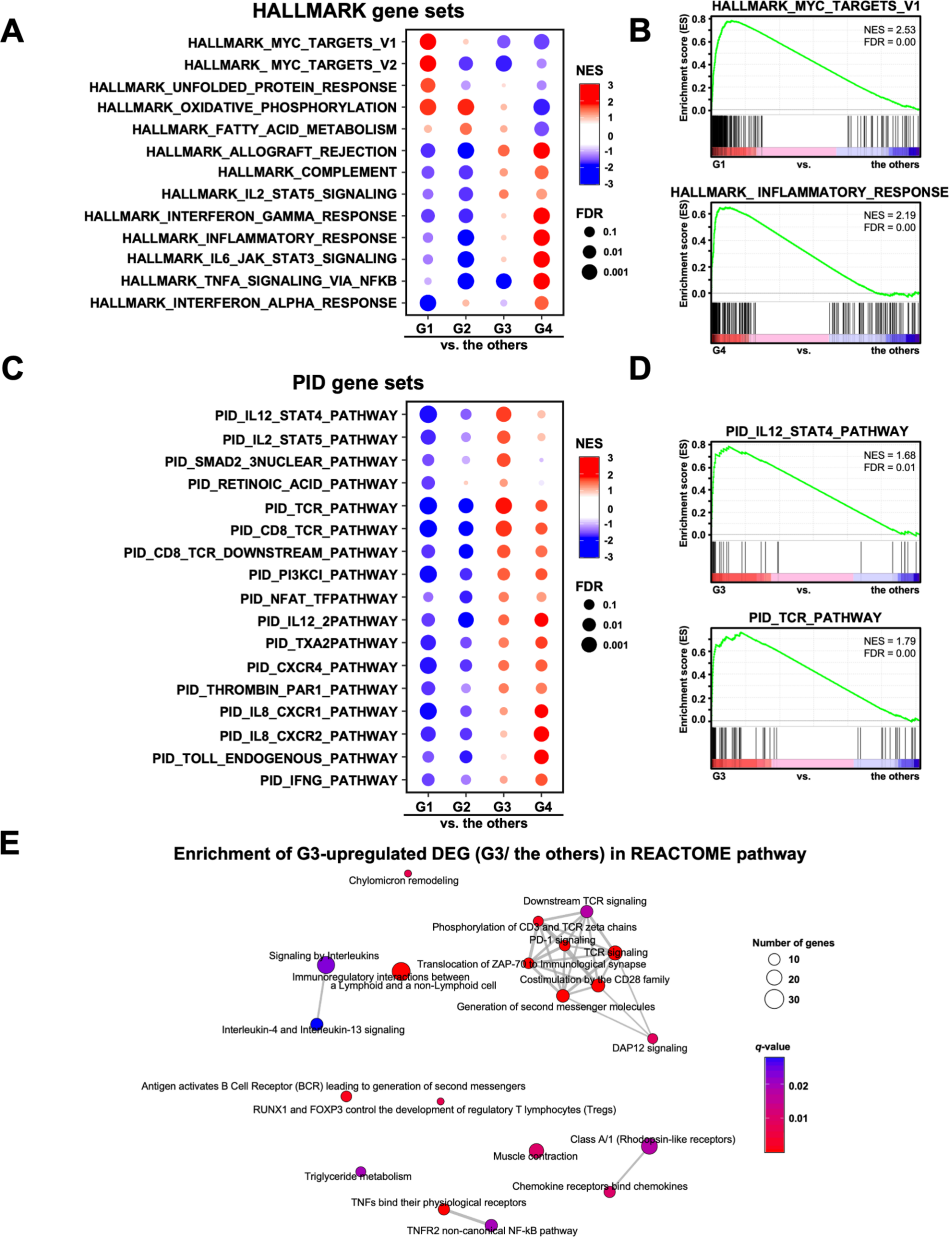
Analysis of the signaling pathways in four transcriptomic groups of patients with gastric cancer. **(A)** Gene Set Enrichment Analysis (GSEA) was performed for each group using the HALLMARK gene sets. Of the 50 HALLMARK gene sets, 13 characteristic gene sets of each group are shown. NES, Normalized Enrichment Score. **(B)** Representative enrichment plots of the HALLMARK MYC_TARGETS_V1 and INFLAMMATORY_RESPONSE gene sets are shown. **(C)** GSEA was performed for each group using the PID gene set. Of the 196 PID gene sets, 17 characteristic gene sets of each group are shown. **(D)** Representative enrichment plots of the PID IL12_STAT4_PATHWAY and TCR_PATHWAY gene sets. **(E)** Enrichment map analysis of the REACTOME pathway was performed on the G3 upregulated DEGs (567 genes).

Next, we performed GSEA on the RNA-Seq data to analyze the pathway characteristics in each group. In the analysis of the HALLMARK gene sets, 13 out of the 50 gene sets in each group were characterized (**Figure 3A**). In G1, the myelocytomatosis (MYC) pathway was activated, although the *MYC* gene itself was not highly expressed (**Figure 3A**). The immune-related pathways (INTERFERON_GAMMA_RESPONSE, INFLAMMATORY_RESPONSE, IL6_JAK_STAT3_SIGNALING, AND TNFA_SIGNALING_VIA_NFKB) were activated in G4, but inactivated in G2 (**Figures 3A, B**). In addition, G3 showed activated immune-related pathways, but to a lower extent than that observed in G4 (**Figure 3A**). We also performed GSEA on the gene sets of the Canonical pathways (BIOCARTA, KEGG_MEDICUS, PID, PEACTOME, WIKIPATHWAYS, and KEGG_LEGACY) (**Supplementary Figure 3A**). Of these, the PID gene sets showed the activated pathways characteristic of the G3 group, such as IL12_STAT4, IL2_STAT5 and TCR pathways (**Figures 3C, D**). In contrast, the IL8_CXCR1/2 pathway was activated in G4. In support of these results, REACTOME pathway analysis of the up-regulated DEGs revealed that TCR signaling and PD-1 signaling were enriched in G3 (**Figure 3E**). These results were consistent with the activation of acquired immunity in G3, as shown using the GO analysis (**Figure 1B**).

**Figure 4:**
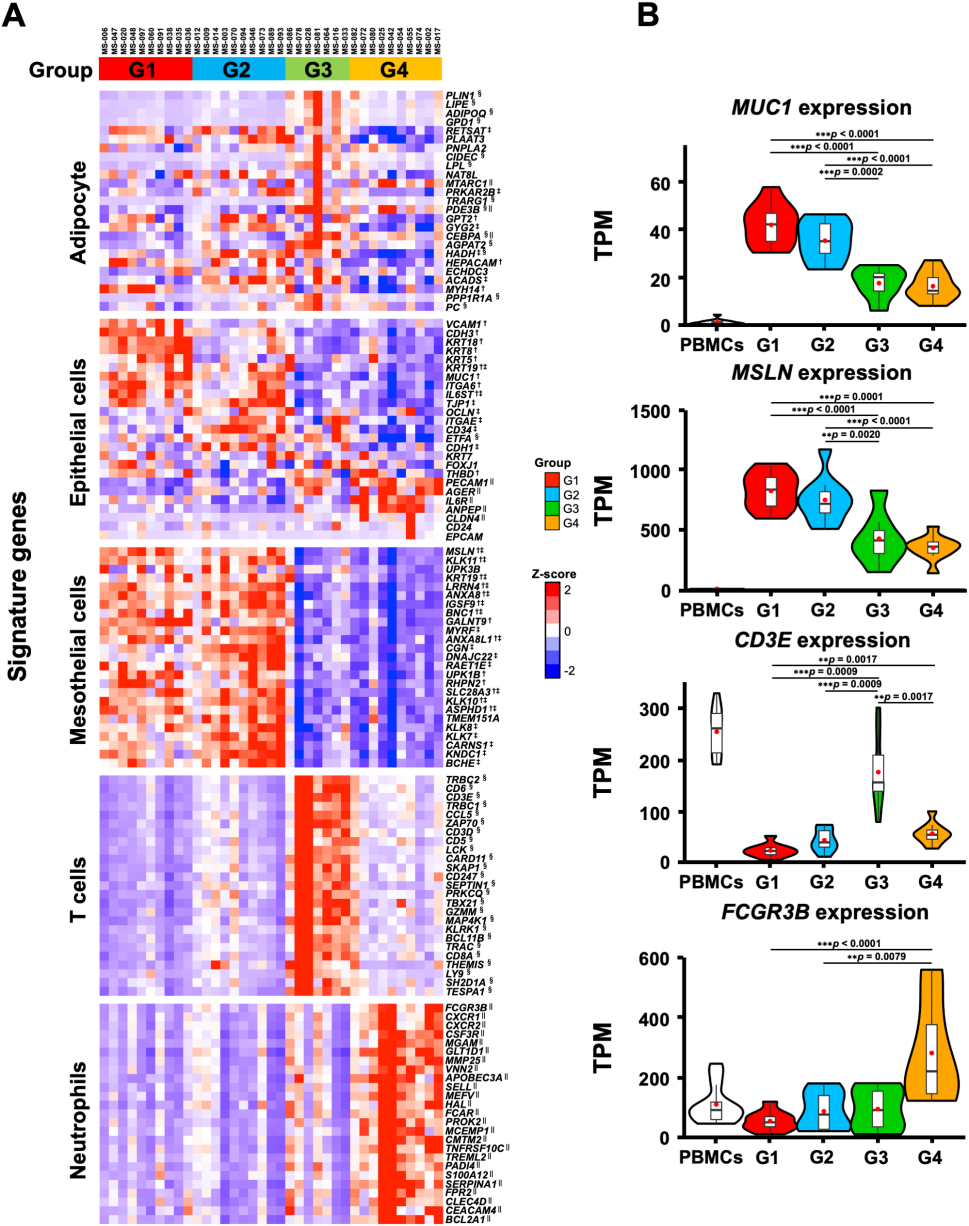
Characteristic gene expression of the cell type signature in the four transcriptomic groups of the patients with gastric cancer. **(A)** The 25 characteristic genes of cell type in each group are shown in the heatmap. Heatmap displayed the z-score of TPM for RNA-Seq. **(B)** Expression of *MUC1*, *MSLN, CD3E*, and *FCGR3B* as specific genes for epithelial, mesothelial, T, and neutrophilic cells, respectively. The RNA-Seq data of PBMCs from healthy subjects are shown as reference. ^**^ *p* < 0.01, ^***^ *p* < 0.001. ^†^, ^‡^, ^§^ and ^||^ indicate significant differences in the G1, G2, G3 and G4 groups.

We also performed a GSEA analysis with cell type signature gene sets, and extracted the top 20 gene sets enriched in each group out of 830 gene sets. **Supplementary Figure 3B** shows 75 gene sets, excluding duplicates, which indicate the characteristic cell type in each group. The G1 and G2 groups were enriched with mesothelial and/or epithelial cells but not with immune cells. In contrast, G3 and G4 were enriched with lymphoid and myeloid cells, respectively. In addition, we examined the expression of the characteristic genes of each cell type in the four transcriptomic groups (**Figure 4**). The genes that characterize the mesothelial or epithelial cells were highly expressed in G1 and G2, whereas those of T cells and neutrophils were enriched in G3 and G4, respectively (**Figure 4A**). For instance, epithelial cell markers such as *MUC1* (53) and mesothelial cell markers such as *MSLN* (54) were highly expressed in G1 and G2, respectively. T cell marker genes such as *CD3E* and *CD3D* (55) were highly expressed in G3, while myeloid cell marker genes such as *FCGR3B* (56) and *S100A12* (57) were highly expressed in G4 (**Figures 4A, B**), which were consistent with the GO analyses showing enrichment of neutrophil degranulation in G4 (**Figure 1B**). These results suggest that the transcriptomic groups of the omental milky spots demonstrated distinctive pathways, gene expression states, and cell types.

### 3.4 Digital cytometric estimation of the immune cells in the omental milky spots and validation using flow cytometry

**Figure 5:**
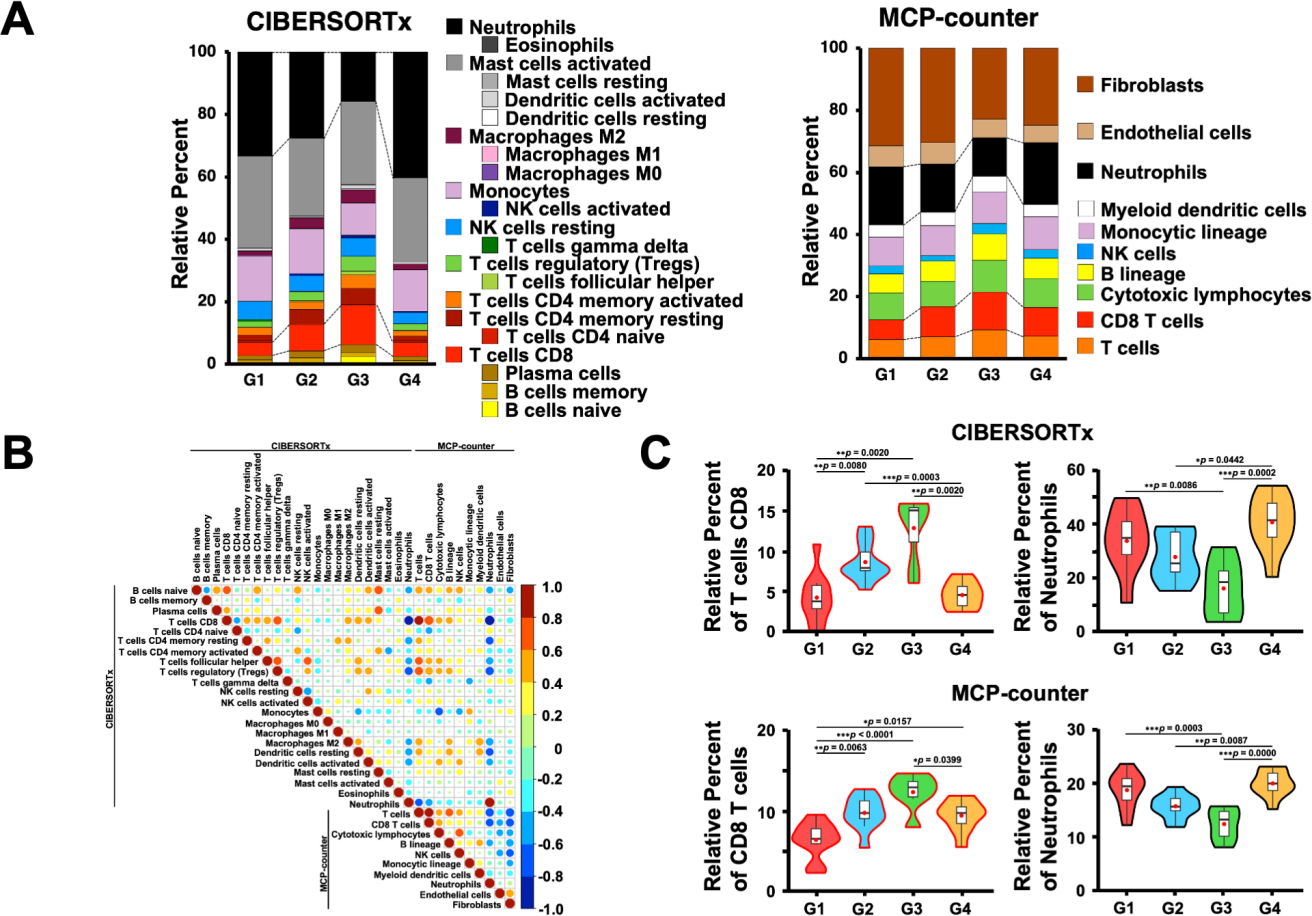
Estimation of immune cells in the omental milky spots of patients with gastric cancer using digital cytometry. **(A)** CIBERSORTx and MCP-COUNTER programs were employed to estimate the immune cells from their RNA-seq data in the omental milky spots of patients with gastric cancer. CIBERSORTx used the LM22 gene signature matrix. The results of immune cell estimation are shown in each group. **(B)** Pearson’s correlation in immune cell estimation results between the two different programs. The correlation coefficient, r > 0.8 was considered as a positive correlation, and r < -0.8 was considered as a negative correlation. **(C)** The percentages of CD8^+^ T cells and neutrophils are shown in each group as representative estimation data. ^*^ *p* < 0.05, ^**^ *p* < 0.01, ^***^ *p* < 0.001.

Since the genes associated with T cells were highly expressed in the G3 group, we first estimated the immune cell frequencies from the RNA-Seq data using two different digital cytometry techniques, CIBERSORTx and MCP-counter (49, 50) (**Figure 5A**). Of note, the frequencies of CD8^+^ T cells and neutrophils were highly correlated between the CIBERSORTx and MCP-counter programs (r ≥ 0.8; **Figure 5B**), and both programs showed that G3 had significantly more CD8^+^ T and B cells and fewer neutrophils than the other groups (**Figure 5C**). In contrast, G4 had more neutrophils than the other groups, which is consistent with the GO and gene expression analysis results (**Figures 1B**, **4**, **5C**).

**Figure 6:**
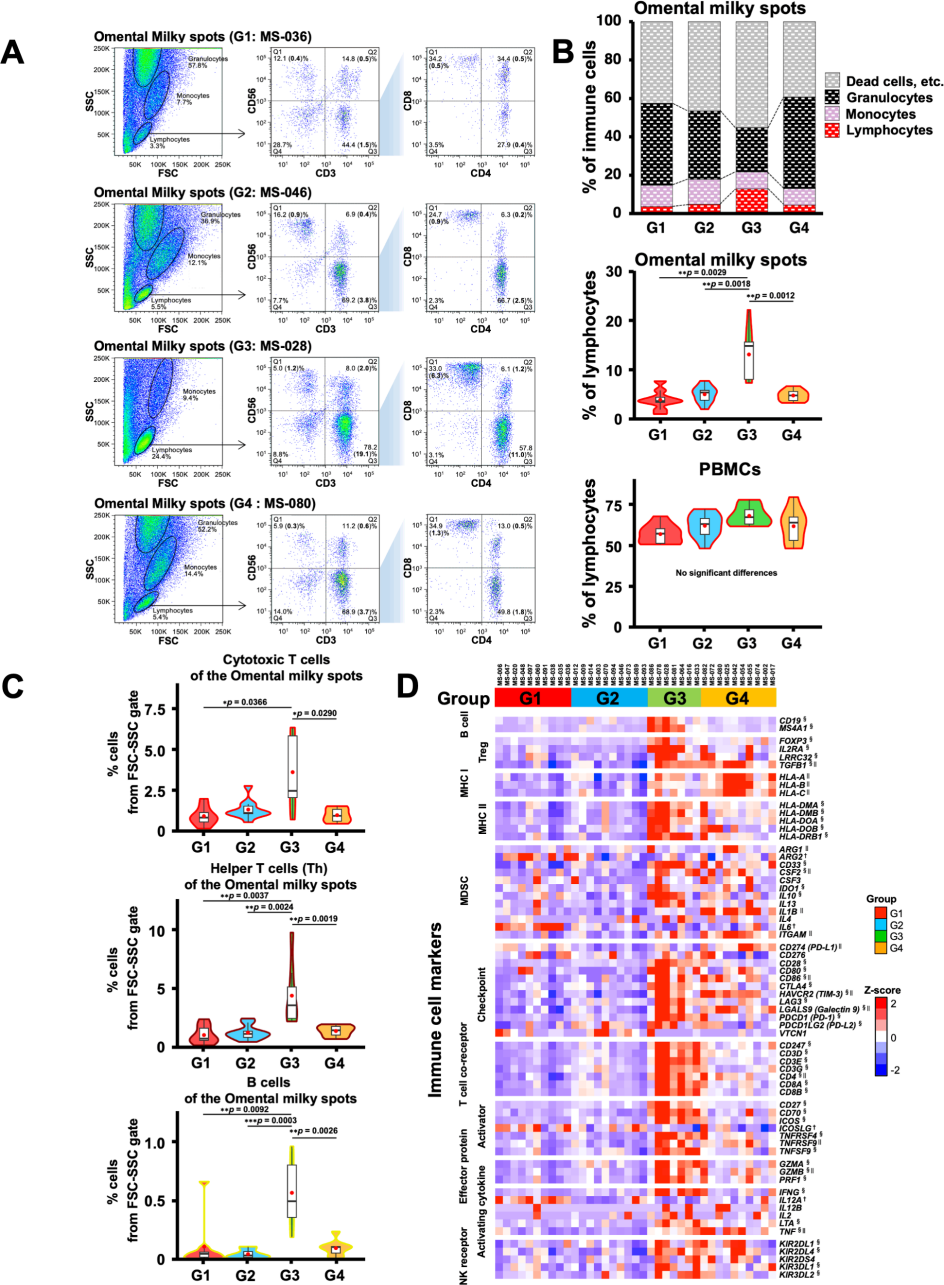
Flow cytometric analysis of immune cells in the omental milky spot from patients with gastric cancer and their characteristics. **(A)** Representative flow cytometry plots are presented for cells of the omental milky spots in each group. The left panel shows cells in the forward scatter-side scatter (FSC-SSC) gate. The middle panel shows the percentage of CD3^+^, CD56^+^, and CD3^+^CD56^+^ cell subsets in the lymphocyte gate. The right panel shows the percentages of CD4^+^, CD8^+^, and CD4^+^CD8^+^ cell subsets in the CD3^+^ cell gate. The bold numbers in parentheses indicate the percentages of cells from the FSC-SSC gate. **(B)** The upper panel shows the percentages of cells of the omental milky spots in the lymphocyte, monocyte, and granulocyte gates in each group. Lymphocytes, monocytes, and granulocytes were verified (**Supplementary Figure 4**). The middle panel shows the percentages of cells of the omental milky spots in the lymphocyte gate in each group. The lower panel shows the percentages of cells in the lymphocyte gate in each group in PBMCs from the same patients. **(C)** The percentages of CD8^+^ cytotoxic T, CD4^+^ helper T, and B cells from the FSC-SSC lymphocyte gate in the omental milky spot in each group. ^*^ *p* < 0.05, ^**^ *p* < 0.01, ^***^ *p* < 0.001. **(D)** Expression of the immune-related marker genes in the omental milky spots in each group is shown in the heatmap. ^†^, ^§^ and ^||^ indicate significant differences in the G1, G3 and G4 groups.

Next, we performed flow cytometry analyses of the cells in the omental milky spots from the same cohort of patients with gastric cancer (**Figure 6A**). G3 showed a higher percentage of cells in the lymphocyte gate, and a lower percentage of cells in the granulocyte gate (**Figure 6B**). In addition, G3 had significantly more T cells, including CD8^+^ T cells (CD3^+^CD8^+^), CD4^+^ T cells (CD3^+^CD4^+^), effector memory CD4^+^ T cells (CD4^+^CCR7^-^CD45RA^-^), and other cell subsets such as B and natural killer cells (**Figure 6C**, **Table 4**). We also performed flow cytometric analysis on PBMCs from the same cohort of patients with gastric cancer (**Supplementary Figure 5**). No statistically significant differences were observed between the four transcriptomic groups with respect to the proportions of immune cells, such as T and B cells, and their subsets in PBMCs (**Supplementary Figure 5C**, **Table 5**). These results demonstrated that the immune cells in PBMCs showed less pronounced differences than those in the omental milky spots.

**Table 4:** Immune cell proportions and statistical analyses in the omental milky spots in each group using flow cytometry.

Cell type	Markers	G1 group	G2 group	G3 group	G4 group	Multiple Comparisons	Pairwise Comparisons					
							G1 vs. G2	G1 vs. G3	G1 vs. G4	G2 vs. G3	G2 vs. G4	G3 vs. G4
		% cells from FSC-SSC gate				*p*-value	*p*-value					
T cells	CD3^+^CD56^-^	2.0466	2.7045	8.6575	2.5175	^**^0.0016	0.5348	^**^0.0036	0.8038	^**^0.0053	0.9977	^**^0.0029
Cytotoxic T cells	CD3^+^CD8^+^	0.8620	1.2604	3.5431	0.9237	^**^0.0099	0.4251	^*^0.0366	0.9906	0.1025	0.5178	^*^0.029
Helper T cells (Th)	CD3^+^CD4^+^	0.9395	1.1451	4.2769	1.2873	^**^0.0010	0.7022	^**^0.0037	0.6196	^**^0.0024	0.8796	^**^0.0019
CD4^+^CD8^+^ T cells	CD3^+^CD4^+^CD8^+^	0.1486	0.1752	0.5374	0.2348	^*^0.0361	0.8618	^*^0.0253	0.8063	0.0599	0.985	0.4654
NKT cells	CD3^+^CD56^+^	0.3143	0.5837	0.9634	0.3375	^*^0.0330	0.2879	0.1003	0.9961	0.6126	0.3969	0.1174
NK cells	CD3^-^CD56^+^	0.4706	0.4450	0.9914	0.4511	^**^0.0077	0.9947	^*^0.0345	0.997	^**^0.0048	1	^*^0.0111
B cells	CD19^+^	0.1035	0.0486	0.5534	0.0861	^***^0.0009	0.9983	^**^0.0092	0.4657	^***^0.0003	0.4408	^**^0.0026
Naïve B cells	IgD^+^CD27^-^	0.0564	0.0229	0.2693	0.0480	^**^0.0023	0.9991	^*^0.0216	0.493	^***^0.0007	0.5148	^*^0.0166
Switched Memory B cells	IgD^-^CD27^+^	0.0132	0.0111	0.1031	0.0145	^*^0.0105	0.9998	^*^0.0334	0.9361	^**^0.0050	0.8417	0.0574
Unswitched B cells	IgD^+^CD27^+^	0.0012	0.0016	0.0126	0.0020	^*^0.0165	0.9947	^*^0.0218	0.6128	^*^0.0409	0.8520	0.1194
CD4^+^ Naïve	CD4^+^CCR7^+^CD45RA^+^	0.0937	0.0363	0.6392	0.0980	^*^0.0126	0.6441	0.0953	0.9906	^*^0.0177	0.1697	0.2658
CD4^+^ Central Memory (CM)	CD4^+^CCR7^+^CD45RA^-^	0.2150	0.1982	1.2434	0.2502	^**^0.0084	1	^*^0.0140	0.9595	^**^0.0050	0.9926	^*^0.0379
CD4^+^ Effector Memory (EM)	CD4^+^CCR7^-^CD45RA^-^	0.7360	1.0369	2.7163	1.0951	^***^0.0005	0.4182	^***^0.0009	0.489	^**^0.0020	0.9999	^**^0.0016
CD4^+^ TEMRA	CD4^+^CCR7^-^CD45RA^+^	0.0296	0.0356	0.1634	0.0673	^*^0.0238	1	^*^0.0191	0.3205	0.0958	0.5733	0.5291
CD8^+^ Naïve	CD8^+^CCR7^+^CD45RA^+^	0.0229	0.0240	0.1570	0.0356	0.0811						
CD8^+^ Central Memory (CM)	CD8^+^CCR7^+^CD45RA^-^	0.0364	0.0628	0.2575	0.0534	^*^0.0318	0.7086	0.0517	0.9363	0.1621	0.9949	0.0591
CD8^+^ Effector Memory (EM)	CD8^+^CCR7^-^CD45RA^-^	0.4550	0.8363	2.2210	0.5702	^**^0.0012	^*^0.0462	^**^0.0094	0.8097	0.1299	0.2891	^**^0.0066
CD8^+^ TEMRA	CD8^+^CCR7^-^CD45RA^+^	0.2870	0.3077	0.9828	0.3455	0.0804						

For Cell type, the multiple comparison tests with G1-4 groups were analyzed by Kruskal-Wallis test (analysis of variance). The pairwise comparison was performed with Steel-Dwass tests when a *P* value for the Kruskal-Wallis test was significant.

Th, Helper T cells; CM, Central Memory; EM, Effector Memory; TEMRA, Terminally differentiated effector memory T cells re-expressing CD45RA. ^*^ *p* < 0.05, ^**^ *p* < 0.01, ^***^ *p* < 0.001. Bold text indicates statically significant.

**Table 5:** Immune cell proportions and statistical analyses in the PBMCs of each group using flow cytometry.

Cell type	Markers	G1 group	G2 group	G3 group	G4 group	Multiple Comparisons
		% cells from FSC-SSC gate				*p*-value
T cells	CD3^+^CD56^-^	31.7257	34.5948	43.9895	31.9611	0.3060
Cytotoxic T cells	CD3^+^CD8^+^	7.1724	9.3197	12.3576	8.8253	0.1410
Helper T cells (Th)	CD3^+^CD4^+^	23.3059	23.5588	29.1971	21.7880	0.7310
CD4^+^CD8^+^ T cells	CD3^+^CD4^+^CD8^+^	0.8047	0.3918	1.2531	0.7455	0.0914
NKT cells	CD3^+^CD56^+^	2.1814	1.7342	3.7719	2.0381	0.5918
NK cells	CD3^-^CD56^+^	8.8566	8.1625	8.9367	6.0399	0.5360
B cells	CD19^+^	2.5123	2.7777	3.8674	1.7825	0.5355
Naïve B cells	IgD^+^CD27^-^	1.6705	1.8492	2.7625	1.1398	0.6563
Switched Memory B cells	IgD^-^CD27^+^	0.5734	0.5893	0.6140	0.4327	0.9621
Unswitched B cells	IgD^+^CD27^+^	0.0791	0.1177	0.1835	0.0750	0.1003
CD4^+^ Naïve	CD4^+^CCR7^+^CD45RA^+^	11.1607	10.8470	11.4613	10.7931	0.9031
CD4^+^ Central Memory (CM)	CD4^+^CCR7^+^CD45RA^-^	9.7012	9.5666	13.7678	8.1402	0.3280
CD4^+^ Effector Memory (EM)	CD4^+^CCR7^-^CD45RA^-^	3.1610	3.4066	4.3173	3.1215	0.4090
CD4^+^ TEMRA	CD4^+^CCR7^-^CD45RA^+^	0.4903	0.6365	0.8952	0.6431	0.3816
CD8^+^ Naïve	CD8^+^CCR7^+^CD45RA^+^	0.6829	1.4714	1.2930	1.5233	0.3658
CD8^+^ Central Memory (CM)	CD8^+^CCR7^+^CD45RA^-^	0.5274	1.0529	1.5649	1.0422	0.0657
CD8^+^ Effector Memory (EM)	CD8^+^CCR7^-^CD45RA^-^	0.8387	1.3661	1.7185	1.1178	0.0977
CD8^+^ TEMRA	CD8^+^CCR7^-^CD45RA^+^	2.6462	2.7345	3.9364	2.3558	0.3460

For Cell type, the multiple comparison tests with G1-4 groups were analyzed by ANOVA or Kruskal-Wallis test (analysis of variance).

Helper T cells; Th, Central Memory; CM, Effector Memory; EM, Terminally differentiated effector memory T cells re-expressing CD45RA; TEMRA.

The above findings were confirmed by the expression of the previously reported marker genes that are characteristic of immune cells (58) in the RNA-Seq data; the marker genes related to T and B cells were higher in the G3 group (**Figure 6D**). The results mentioned above confirmed that the G3 group, which has a good prognosis pattern and had more T cells and B cells than the other groups in the omental milky spots.

### 3.5 Identification of potential prognostic marker genes in the omentum related to DEGs of G3

**Figure 7:**
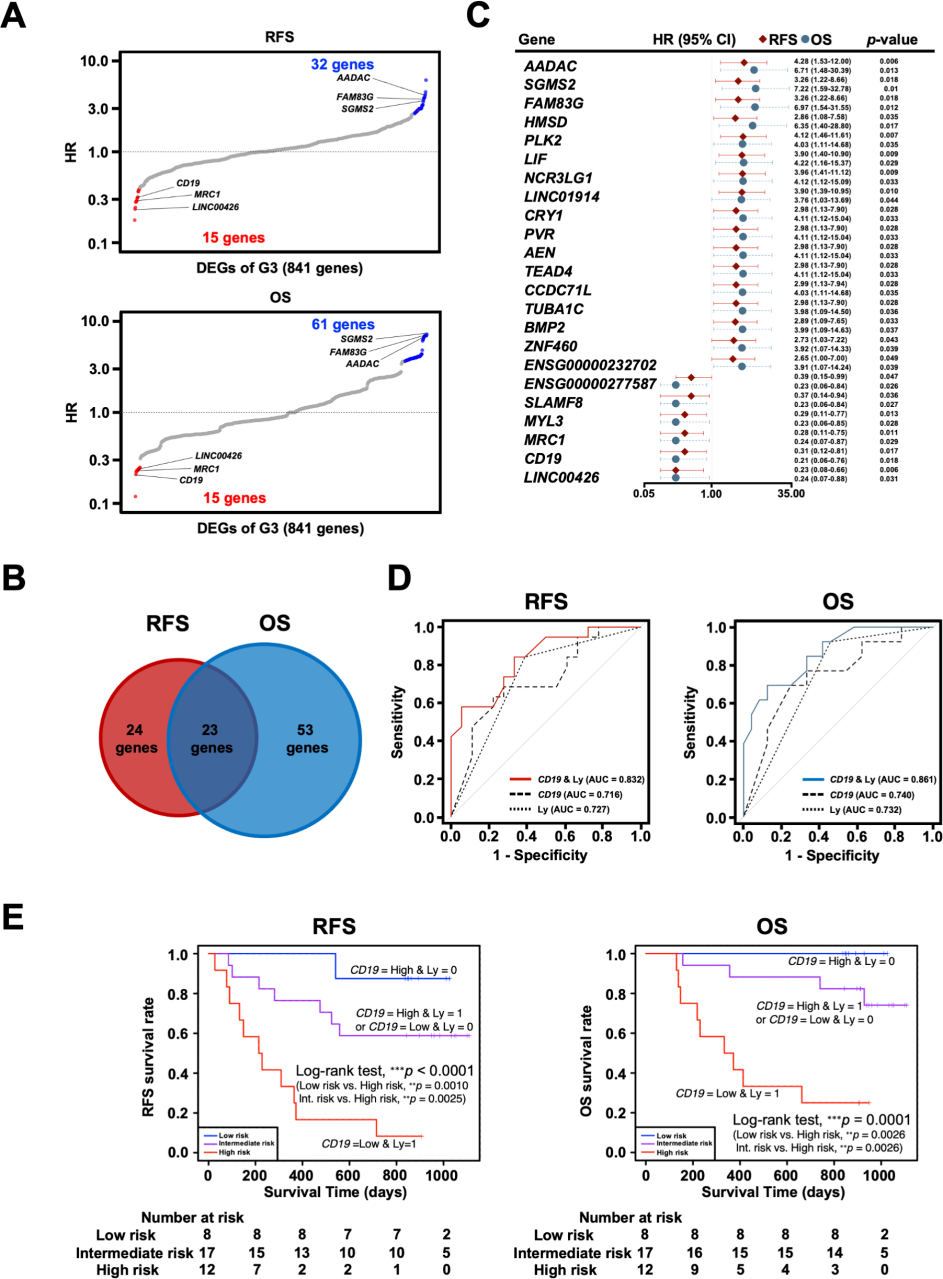
Exploration and identification of potential prognostic marker genes in the omentum of patients with gastric cancer. **(A)** Univariate Cox regression analysis for RFS and OS with DEGs of G3. Red dots indicate G3-upregulated DEGs with Cox regression *p*-value < 0.05, and blue dots indicate G3-downregulated DEGs with Cox regression p-value < 0.05. **(B)** Venn diagram of overlap between prognosis-related genes for RFS and OS in Cox regression analysis. **(C)** Forest plot of 23 potential prognostic genes common to RFS and OS, in which the hazard ratio (HR), corresponding 95% confidence intervals, and p-values are shown. **(D)** Survival ROC curve analysis of potential prognostic predictors, comparing the predictive accuracy of models for *CD19* alone, Ly alone, and the combination of both variables. ROC, Receiver operating characteristic; AUC, The area under the curve. **(E)** Kaplan-Meier survival curve analysis of RFS and OS for high (CD19-low and Ly1)-, intermediate (CD19-low and Ly0 or CD19-high and Ly1)- and low (CD19-high and Ly0)-risk groups. ^**^ *p* < 0.01, ^***^ *p* < 0.001.

To further investigate the clinical significance of genes specific to the G3 group, univariate Cox proportional hazards regression analysis was performed to search for genes associated with prognosis: 47 genes for RFS and 76 genes for OS were identified as genes with *p*-values < 0.05 in Cox regression (**Figure 7A**). As 23 genes were shared between them, they were considered potential prognostic genes in the omentum (**Figures 7B, C**). In addition, a multivariate Cox proportional hazards analysis using these 23 prognostic genes and the clinicopathological factors, Ly and pN, that showed statistical significance (**Table 3**) was performed to identify combinations of variables with high predictive accuracy. The combination of the B cell marker *CD19* and Ly, in particular, showed improved prognostic accuracy compared to a single variable (area under curve of RFS = 0.832 and area under curve of OS = 0.861) (**Figure 7D**). Kaplan-Meier analysis also showed that the high-risk group with low *CD19* and Ly1 had a significantly worse prognosis in terms of RFS (*p* < 0.0001) and OS (*p* = 0.0001) (**Figure 7E**).

## 4 Discussion

The omental milky spots are major implantation sites of malignant cells in peritoneal dissemination. Previous studies have reported the characteristics of omental milky spots in several human cancers, including ovarian and colorectal cancers with metastasis in the omentum (6, 59, 60). However, only a few have reported data concerning the omentum in gastric cancer, although it is likely that the immune cells in the omental milky spots may function differently depending on the cancer types and stages. In this study, we investigated the gene expression profiles in the cells from the omental milky spots in patients with gastric cancer and their association with clinical information. To our knowledge, this is the first report to demonstrate that the immune microenvironment in the omental milky spots may vary significantly depending on the stages of tumor progression in gastric cancer.

In this study, the transcriptomic analysis of the cells in the omental milky spots revealed that the patients were classified into four groups (G1–4) according to their gene expression profiles (**Figure 1A**). Notably, the G3 group was characterized by high expression of genes related to adaptive immune cells, including T and B cells. In addition, it included more patients with macroscopic type 0, Ly0, V0, and pStage I and showed a better prognosis. Therefore, tumor progression, which facilitates Ly and/or V, may be associated with the compositions and proportions in the omental milky spots. We have previously reported that Ly is an independent prognostic factor in gastric cancer and tends to worsen the prognosis, especially in advanced cancer with lymph node metastasis (61). Based on our results, lymphatic infiltration may result in a decrease in the adaptive immune cells in the omental milky spots and facilitate further tumor progression. Future studies are warranted to confirm this hypothesis.

In contrast to the G3 group, the other groups (G1, G2, and G4) showed immune-suppressive environments in the omental milky spots and poor prognosis. Notably, the G4 group was characterized by enrichment of genes related to neutrophil degranulation and innate immunity in the omental milky spots. Neutrophils have immunosuppressive functions in various types of cancer, including gastric cancer (62). For example, activated neutrophils form a pre-metastatic niche by extruding chromatin webs called neutrophil extracellular traps and providing a favorable environment for metastatic cells (63). In addition, G4 showed fewer lymphocytes, suggesting an impaired anti-tumor immunity. In contrast, in the G1 group, the MYC pathway and ribosome biogenesis were activated (**Figures 1B**, **3A**). KRT18, which has been reported to promote migration and invasion in gastric and colorectal cancer (64, 65), was also highly expressed (**Figure 4A**). In the G2 group, the metabolism and degradation of branched-chain amino acids (BCAAs), including valine, leucine, and isoleucine, were activated (**Supplementary Figure 3A**). Since BCAAs deficiency has been suggested to promote tumor metastasis (66), the G2 group may have a tumor-promoting environment. Regarding the common features of the G1 and G2 groups, the transcriptome analyses revealed that neither the innate nor adaptive immune-related genes were enriched in the omental milky spots, suggesting immune-desert microenvironment. In contrast, cell type signatures of the mesothelial and/or epithelial cells were highly expressed in the G1 and G2 groups. Recently, it has been reported that mesothelial cell-derived cancer-associated fibroblasts form a microenvironment that promotes cancer progression, and mesothelial cells may be involved in the decrease in the immune cells (24–28). We did not analyze the pathologically metastatic omentum tissues in this study; however, the high expression of genes that are characteristic of epithelial cells in the G1 and G2 groups suggests that the cancer cells may have already metastasized to the omentum at a level that cannot be pathologically confirmed. Notably, G1 tended to have a poorer prognosis than the other groups, although the differences were not statistically significant. Since immune suppressive genes, such as IL-6 and arginase 2, were significantly enriched in G1, they may also be associated with a worse prognosis in G1 (**Figure 6D**).

In the present analysis, the four groups classified using the transcriptomic analysis differed significantly in the proportions of immune cells, such as T cells and neutrophils, in the omental milky spots, but no statistically significant differences were observed in PBMCs (**Tables 4**, **5**). Considering our hypothesis that tumor progression, such as Ly and V, affects the immune microenvironment in the omentum, it may be reasonable that the immune cells in the omental milky spots fluctuate more sensitively than those in PBMCs. Although the milky spots contribute to the peritoneal seeding of cancer cells by acting as a gate through the abdominal cavity, the potential impact of the complete resection of the greater omentum on preventing recurrence and improving survival after gastrectomy in gastric cancer is controversial. This study suggested that the immune microenvironment in the omentum may be affected substantially by the tumor stages; thus, the indication of omentectomy during gastrectomy may differ accordingly. For example, omentectomy should not be performed in earlier tumor stages without Ly or V, which is expected to show an anti-tumor immune microenvironment in the omentum. In contrast, complete resection of the omentum may be recommended in advanced tumor stages with immunosuppressive microenvironment in the omentum.

Measuring immune cell subsets and analyzing gene expression in the omentum is a higher hurdle than in PBMCs, but may be a more accurate predictor of recurrence and prognosis. Indeed, of the 23 potential prognostic genes of the omentum that we identified, many genes have been reported to be prognostically relevant in other cancers (67–70). Notably, multivariate Cox proportional hazards analysis demonstrated that the combination of the B cell marker CD19 and Ly showed improved predictive accuracy, suggesting the prognostic importance of analyzing gene expression in the omentum. Given the critical role of B cells in anti-tumor immunity (71, 72), it may be possible and important to translate our findings into clinical applications. Since the sample size in this study was relatively small, this model needs to be validated or optimized in further studies with larger numbers of patients. Nevertheless, if validated, we believe that this model can be applied in real clinical settings to predict patients who are likely to have earlier recurrence and/or poor prognosis and should be treated with postoperative adjuvant therapies. To increase this model’s practicality, simplified methods such as reverse transcriptase-polymerase chain reaction (RT-PCR) to assess CD19 expression could be developed to reduce cost and time. Currently, many prognostic models have been established using multiple genes assessed by RNA-Seq or other methods to improve the accuracy of prognosis prediction. However, if a simplified method for assessing a single or a small number of genes is sufficient for prognosis prediction, it will facilitate its clinical implementation.

In summary, our study revealed that the immune cells in the omental milky spots of patients with gastric cancer could be divided into four groups according to their gene expression profiles. Patients in the G3 group, who had earlier tumor stages and a better prognosis, showed an immune-inflamed microenvironment with more adaptive immune cells, such as T and B cells. As most patients in the G3 group did not show Ly or V, it is possible that lymphatic and venous infiltration of tumor cells affects the composition and proportion of immune cells in the omentum, and the results of this study could be applied to predict patient prognosis in the future.

This study had some limitations. First, the sample size was relatively small because this study was conducted as an exploratory study to show the potential importance of omental milky spots in gastric cancer, which has not been addressed in previous studies. This may have affected the study’s statistical power and ability to detect more subtle differences or associations. In addition, unfortunately, there are few reports on transcriptome analysis of the omentum of patients with gastric cancer, which makes it difficult to validate the predictive model constructed from the study’s results with data from public databases and other sources. Therefore, further studies with larger sample sizes are needed to increase the generalizability and robustness of the results with complex biological systems and multiple variables. Furthermore, this study included patients who received neoadjuvant immunotherapy or chemotherapy before surgery, but it is possible that these may affect the immune cells in omental milky spots. Therefore, it would be important to compare the characteristics of omental milky spots between patients with and without neoadjuvant treatments such as immunotherapy and chemotherapy. In the future, follow-up of clinical information should be continued and the number of clinical samples should be increased to gain a more comprehensive understanding of the immune status and clinical role of the omentum and to improve the accuracy of prediction.

## Data Availability

The original contributions presented in the study are included in the article/**Supplementary Material**, further inquiries can be directed to the corresponding author.
