# Supplementary material for: Characteristics and clinical significance of immune cells in omental milky spots of patients with gastric cancer
Source: Front Immunol. 2025 Jan 30;16:1521278. doi: 10.3389/fimmu.2025.1521278 (PMC11821591; doi:10.3389/fimmu.2025.1521278)
Supplement: Supplementary file 2 [file DataSheet2.docx]

Supplementary Material

# Supplementary Data

The RNA-Seq datasets presented in the study are included in the article/Supplementary Material.

# Supplementary Figures

**Supplementary Figure 1. Validation of four transcriptomic groups using the differentially expressed gene (DEG) analysis.**

(A) Distribution of the numbers of DEGs for each comparison. DEGs were extracted under the condition that |log_2_FC| ≥ 1, *q* < 0.05. (B) Volcano plot of DEGs between G3 and the other groups (combined). Upregulated (log_2_FC ≥ 1, *q* < 0.05) and downregulated (log_2_FC ≤ -1, *q* < 0.05) DEGs are indicated in red and blue dots, respectively. (C) GO analysis was performed on the upregulated DEGs for each group, and the top 10 terms are shown.

**Supplementary Figure 2. Association between clinical information and prognosis (relapse-free survival (RFS) and overall survival (OS)) in the patients with gastric cancer.**

(A) Kaplan–Meier survival curves of relapse-free survival (RFS) and overall survival (OS) in the groups without or with lymphatic invasion (ly = 0 vs. ly = 1a, 1b, 1c). (B) Kaplan–Meier survival curves of RFS and OS in the groups without or with lymph node metastasis (pN = 0 vs. pN ≥ 1). (C) Kaplan–Meier survival curves of RFS and OS in the groups without or with distant metastasis (pM = 0 vs. pM = 1). *P* values were calculated using log-rank test. ^*^*p* < 0.05; ^**^*p* < 0.01.

(Continued to the next page)

**Supplementary Figure 3. Characteristic canonical pathways and cell-type signature gene sets in the four transcriptomic groups of patients with gastric cancer.**

(AB) GSEA was performed for each group using the canonical pathway and cell-type signature gene set. Of the 3494 canonical pathways and 830 cell-type signature gene sets, respectively, the top 20 gene sets in each group were selected and 77 and 75 gene sets were shown excluding duplicates.

**Supplementary Figure 4. Characterization of cells in the lymphocyte, monocyte, and granulocyte gates using flow cytometry.**

(A) Representative flow cytometry results of cells in the gates of lymphocytes, monocytes, and granulocytes. Cells in the lymphocyte, monocyte, and granulocyte gates in the FSC-SSC gate were stained for monocyte marker (CD14) and monocyte and granulocyte markers (CD11b and CD33) and analyzed by flow cytometry.

**Supplementary Figure 5. Flow cytometric analysis of immune cells in Peripheral blood mononuclear cells (PBMCs) of patients with gastric cancer and their proportions in each group.**

(A) Representative flow cytometry results are presented in PBMCs of the same patients in each group. The left panel shows the cells in the forward scatter-side scatter (FSC-SSC) gate. The middle panel shows the percentages of CD3^+^, CD56^+^, and CD3^+^CD56^+^ cell subsets in the lymphocyte gate. The right panel shows the percentages of CD4^+^, CD8^+^, and CD4^+^CD8^+^ cell subsets in the CD3^+^ gate. The bold numbers in parentheses indicate the percentages of cells in the FSC-SSC gate. (B) The percentages of cells in the lymphocyte, monocyte, and granulocyte gates in PBMCs of each group. (C) The percentages of CD8^+^ and CD4^+^ T cells in the FSC-SSC gate in PBMCs of each group.
